# Effect of maternal diabetes on female offspring

**DOI:** 10.1590/S1679-45082014AO3200

**Published:** 2014

**Authors:** Juliana de Oliveira Martins, Maurício Isaac Panício, Marcos Paulo Suehiro Dantas, Guiomar Nascimento Gomes

**Affiliations:** 1 Universidade Federal de São Paulo, São Paulo, SP, Brazil.

**Keywords:** Pregnancy, animal, Fetal development, Diabetes mellitus, experimental, Hypertension, Urea, Creatinine, Streptozocin/administration & dosage

## Abstract

**Objective:**

To evaluate the effect of maternal diabetes on the blood pressure and kidney function of female offspring, as well as if such changes exacerbate during pregnancy.

**Methods:**

*Diabetes mellitus* was induced in female rats with the administration of streptozotocin in a single dose, one week before mating. During pregnancy, blood pressure was measured through plethysmography. On the 20^th^ day of pregnancy, the animals were placed for 24 hours in metabolic cages to obtain urine samples. After the animals were removed from the cages, blood samples were withdrawn. One month after pregnancy, new blood and urine sample were collected. Kidney function was evaluated through proteinuria, plasma urea, plasma creatinine, creatinine excretion rate, urinary flow, and creatinine clearance.

**Results:**

The female offspring from diabetic mothers showed an increase in blood pressure, and a decrease in glomerular filtration rate in relation to the control group.

**Conclusion:**

Hyperglycemia during pregnancy was capable of causing an increase in blood pressure and kidney dysfunction in the female offspring.

## INTRODUCTION

Modifications during embryonic development have been associated with the development of arterial hypertension and alterations in renal function in experimental models, in which changes, such as intrauterine nutrition, *diabetes mellitus* induction, and sleep deprivation, were imposed during the period of pregnancy.^(1-3 )^

In experimental models that mimic gestational diabetes or type 1 diabetes, it was noted that the offspring of diabetic mothers, when adults, present arterial hypertension and a lower glomerular filtration rate, as well as a reduced capacity to excrete sodium when submitted to an overload of this ion.^(2,4-6) ^Additionally, the study of the renal cortex showed an increase in expression of epithelial sodium channels (ENaC) and sodium/potassium ATPase (Na^+^/K^+^ATPase), with no reduction of the sodium/hydrogen exchanger (NHE3) or other sodium transporters, suggesting that sodium retention was due to the increased reabsorption of sodium in distal segments of the nephron. Recently, Aceti et al.^(7 )^ performed a systematic review and meta-analysis of studies reporting arterial hypertension in children born from diabetic mothers, confirming that children of diabetic mothers have higher levels of blood pressure (BP) than controls.

In experimental models it was shown that exposure to hyperglycemia during the fetal period hinders nephrogenesis.^( 4 ) ^Prior studies in our laboratory showed that male offspring of rats with induced *diabetes mellitus * developed early arterial hypertension, with no change in number of nephron. In these animals, the decline of the endothelium-dependent vasodilation response seen in the mesenteric bed seems to contribute towards the development of arterial hypertension. L-arginine supplementation for the animals was capable of preventing hypertension, confirming the participation of the nitric oxidesystem in this experimental model.^(8) ^ However, most of the studies were performed in male animals.

Increased BP is the main risk factor for cardiac diseases, stroke, and kidney diseases. It is estimated that one in every six people in the world is hypertensive and this number is expected to rise to 1.5 billion in 2025.^(9) ^In Brazil, the prevalence of hypertension is between 5 and 30%, depending on the area studied.^(10) ^Although the prevalence of arterial hypertension is greater in men, it is not insignificant in women. It is believed that about 30% of the women in the world’s population are hypertensive.^(11) ^The increase of obesity in young women in the developed countries is also an alarming fact, since it should contribute to the increase in incidence of diabetes in pregnant women.

## OBJECTIVE

To evaluate if females from the offspring of diabetic mothers present with modifications in kidney function and arterial hypertension, besides verifying if these alterations are more marked during pregnancy.

## METHODS

Male and female Wistar rats, obtained at the *Centro de Desenvolvimento de Modelos Experimentais para Medicina and Biologia da Universidade Federal de São Paulo* (UNIFESP), were housed in collective plastic cages and maintained with free access to feed and water. To get pups of diabetic mothers, *diabetes mellitus* was induced in the female Wistar rats with the administration of a single dose of streptozotocin (60mg/kg ip.) one week before mating. Two days after the administration of streptozocin, blood sugar levels were determined with the Advantage II device (Boehringer, Mannheim, Germany) from the caudal vein blood. Diabetic rats were considered those with blood sugar levels ≥250mg/dL. After the pups were born, both from the controls and the diabetic mothers, the offspring was reduced to six pups that remained with the mothers until they were weaned (at 28 days). After this step, the pups were place in collective cages. At 2 months and 15 days of age, the animals were trained to enter a containment cylinder for the measurements of BP using the indirect method of plethysmography. After obtaining the stable values of BP, the females were allowed to mate. Day 1 of gestation was considered the day when pregnancy was confirmed by vaginal smear. The BP values were also obtained throughout pregnancy.

On the 20^th^ day of pregnancy, the animals were placed for 24 hours in metabolic cages (Criffa, Barcelona, Spain), to collect urine samples to determine creatine excretions and proteinuria. After removing the animals from the metabolic cages, blood samples were collected to measure creatinine and urea levels. One month after the birth of the pups, the rats were placed again into metabolic cages for new measurements of kidney function, and then euthanized.

This project was carried out during the period from July 2013 to July 2014, with approval number 0048/12 from the Research Ethics Committee of UNIFESP. The Informed Consent Form was not applied.

### Renal function study

To study the kidney function, plasma concentrations of urea and creatinine were assessed. The creatinine clearance was calculated as an index of glomerular filtration rate. Plasma and urine concentrations of creatinine were determined by the colorimetric method. The following formula was used to calculate clearance:


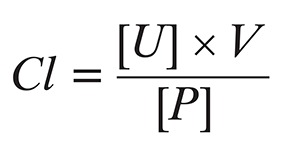


Where Cl indicates creatinine clearance; U, urinary creatinine concentration; P, plasma concentration of creatinine; and V, urinary flow.

The method used to determine proteinuria was sulfosalicylic acid precipitation.

### Morphological study

The kidneys of the female controls and pups of non-pregnant diabetic mothers at 3 months were dissected, freed of connective tissue, weighed, cut longitudinally, and fixed in Bouin. Histologic slices of 5μm were made, which were then stained using the hematoxylin-eosin and Verhoeff. The images were acquired on a Nikon^®^ microscope connected to a microcomputer. Analyses of the images were single-blinded.

From each slide stained by hematoxylin-eosin, 20 sequential images (fields) were obtained, starting at the hilus and covering the renal cortex, with analysis of the glomerular area and the number of glomeruli per field.

In order to evaluate arteriolar thickness, on each slide stained as per Verhoeff, 12 arterioles were analyzed, from which four measurements of thickness per arteriole were obtained.

### Statistical analysis

For the statistical analysis, the non-paired *t*-test and one-way variance analysis (one-way ANOVA) were used, followed by Bonferroni test. A value of p≤0.05 was adopted as statistically significant. The results were presented as mean±standard error.

## RESULTS

**Figure 1:**
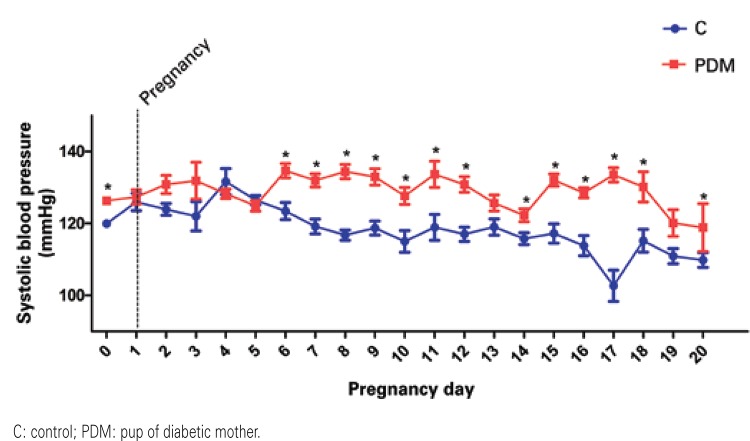
Systolic blood pressure (in mmHg) along the period of pregnancy in the control and pup of diabetic mother groups. p≤0.05 *versus* C

Table 1 shows the mean systolic blood pressure values measured during pregnancy in the control and diabetic mother offspring groups, as well as the mean BP this period ( Figure 1 ).

**Table 1:** Mean values of systolic blood pressure (in mmHg) measured throughout the experimentation period in the control and diabetic offspring groups

Pregnancy day	Control	Pups from diabetic mothers
Before pregnancy	119.98±1.07 (44/11)	127.39±1.09 (55/13)
Day 1	125.97±2.40 (33/11)	130.85±1.98 (38/10)
Day 2	123.92±1.65 (24/7)	131.90±2.51 (40/10)
Day 3	122.00±4.08 (7/2)	128.25±5.12 (20/5)
Day 4	131.63±3.62 (8/2)	124.90±1.33 (8/2)
Day 5	126.50±1.28 (26/8)	134.68±1.47 (20/5)
Day 6	123.44±2.38 (27/8)	131.97±2.00 (39/11)
Day 7	119.17±2.06 (30/10)	134.42±1.86 (39/10)
Day 8	116.75±1.45 (32/10)	132.96±2.00 (30/10)
Day 9	118.72±1.93 (25/7)	127.67±2.28 (26/7)
Day 10	115.00±3.03 (12/4)	133.67±2.35 (21/6)
Day 11	118.93±3.58 (14/4)	130.84±3.63 (15/4)
Day 12	117.00±1.99 (27/8)	125.70±2.18 (31/8)
Day 13	119.00±2.27 (22/7)	122.26±2.21 (37/10)
Day 14	115.76±1.64 (29/8)	132.06±1.79 (35/10)
Day 15	117.21±2.66 (34/10)	128.52±1.70 (40/11)
Day 16	113.84±2.80 (19/6)	133.52±1.41 (27/10)
Day 17	102.67±4.33 (3/1)	130.17±2.00 (14/7)
Day 18	115.18±3.18 (11/3)	120.13±4.19 (12/4)
Day 19	110.93±2.10 (28/8)	118.88±3.68 (16/7)
Day 20	109.84±2.06 (25/7)	127.39±6.67 (12/6)
General mean	118.26	128.91

Values presented as mean±standard error (number of measurements/number of animals).

**Figure 2:**
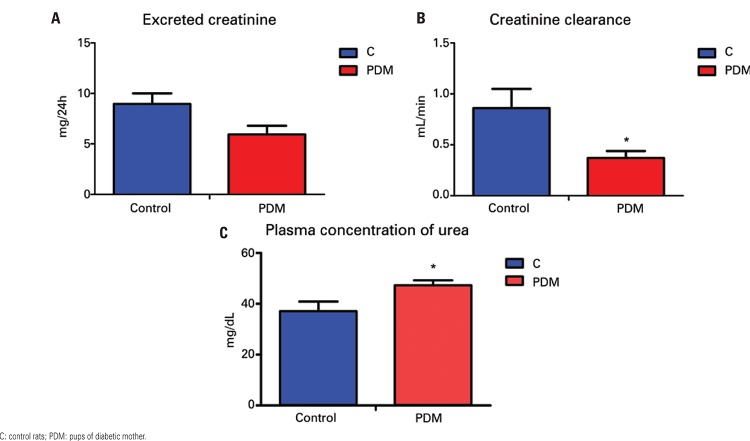
Kidney function of control rats and pups of diabetic mother on the 20th day of pregnancy. (A) Excreted creatinine value; (B) Creatinine clearance; (C) plasma concentration of urea. p≤0.05 *versus* C

The kidney function parameters on the 20^th^ day of pregnancy showed that the group of pups from diabetic mothers had reduced creatinine clearance and increased plasma concentration of urea, but there was no significant reduction in the excreted creatinine value ( Figure 2 and Table 2 ).

**Table 2:** Kidney function parameters on the 20th day of pregnancy

	Proteinuria (mg/24h)	Plasma urea (mg/dL)	Plasma creatinine (mg/mL)	Excreted creatinine rate (mg/24h)	Creatinine clearance (mL/min)	Urinary flow (mL/24h)
Controls	2.17±0.38 (11)	37.09±3.78 (13)	1.20±0.25 (10)	8.96±1.05 (15)	0.86±0.19 (10)	11.70±0.87 (15)
Pups of diabetic mothers	1.95±0.37 (11)	47.26±1.9* (14)	1.02±0.10 (9)	5.93±0.87 (10)	0.37±0.07* (8)	11.90±0.62 (15)

*p≤0.05 *versus *control. Values presented as mean ± standard error (n).

**Figure 3:**
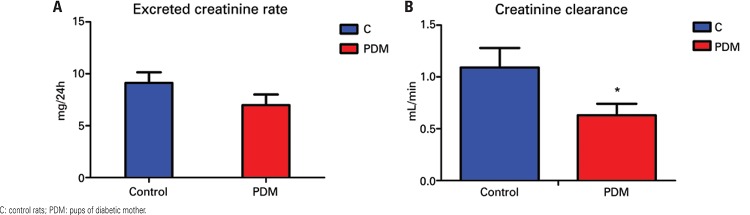
Kidney function in control rats and pups of diabetic mothers one month after pregnancy. (A) Excreted creatinine rate; (B) creatinine clearance. * p≤0.05 *versus* control

Kidney function was reevaluated after one month, and also showed reduced creatinine clearance in female pups of diabetic mothers ( Figure 3 and Table 3 ).

**Table 3:** Kidney function parameters one month after pregnancy

	Proteinuria (mg/24h)	Plasma urea (mg/dL)	Plasma creatinine (mg/mL)	Excreted creatinine rate (mg/24h)	Creatinine clearance (mL/min)	Urinary flow (mL/24h)
Controls	0.93±0.28 (9)	53.85±5.84 (12)	0.74±0.13 (12)	9.12±1.02 (12)	1.09±0.19 (12)	11.99±0.99 (12)
Pups of diabetic mothers	0.96±0.27 (11)	41.33±1.95 (12)	0.86±0.07 (12)	6.98±1.02 (12)	0.63±0.11* (12)	11.50±0.84 (12)

*p≤0.05 *versus *control. Values presented as mean ± standard error (n).

**Figure 4:**
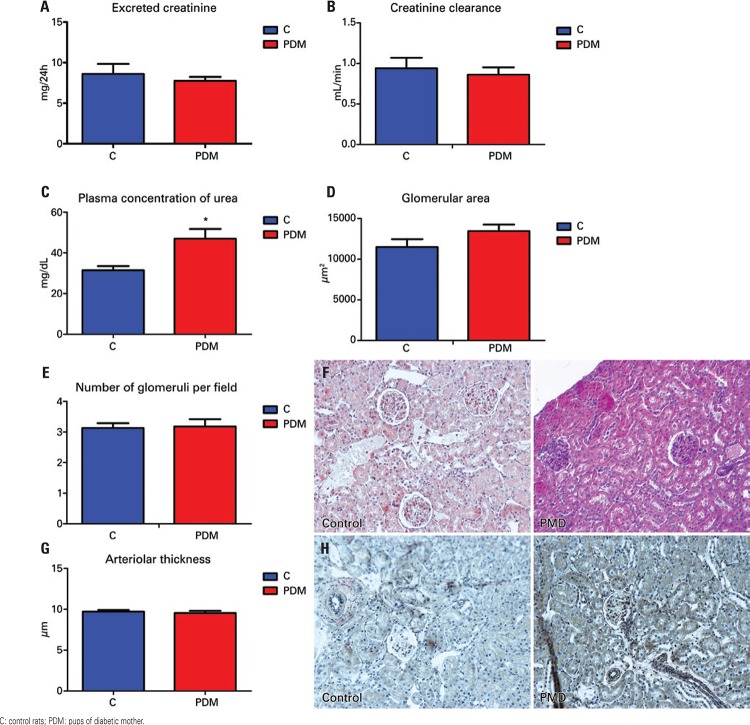
Control rats and pups of non-pregnant diabetic mother at three months. Kidney function: (A) excreted creatinine rate; (B) creatinine clearance; (C) plasma urea. Kidney morphology: (D) glomerular area; (E) number of glomeruli per acquired field; (F) photomicrographs of histologic slices stained with hematoxylin and eosin; (G) arteriolar thickness; (H) photomicrographs of histologic slices as per Verhoeff. *p≤0.05 *versus *control

Comparing control rats and pups of non-pregnant diabetic mothers at 3 months, kidney function compromise was evident by the increased plasma concentration of urea, which as was mentioned before, is exacerbated by the pregnant state ( Figures 4A, 4B, 4C and Table 4 ).

**Table 4:** Kidney function parameters of non-pregnant rats at three months

	Proteinuria (mg/24h)	Plasma urea (mg/dL)	Plasma creatinine (mg/mL)	Excreted creatinine rate (mg/24h)	Creatinine clearance (mL/min)	Urinary flow (mL/24h)
Controls	1.17±0.28 (4)	31.45±2.10 (8)	0.80±0.15 (5)	8.61±1.24 (8)	0.94±0.13 (5)	7.96±1.09 (9)
Pups of diabetic mothers	0.68±0.11 (2)	46.98±4.7* (6)	0.67±0.08 (6)	7.76±0.48 (7)	0.86±0.09 (6)	9.50±0.51 (7)

*p≤0.05 *versus *control. Values presented as mean ± standard error (n).

Kidney morphometric analysis with hematoxylin-eosin showed no statistical difference in glomerular area and in the numbers of glomeruli in these rats at 3 months (Figures 4D, 4E, and 4F). The evaluation of arteriolar thickness as per Verhoeff also revealed no difference between the groups (Figures 4G and 4H).

## DISCUSSION

In the present study, it was observed that the female offspring developed pre-hypertension, and even without showing high pressure levels such as the male gender, the rats exposed to intrauterine hyperglycemia already displayed early alterations in renal function, which became more evident with the pregnancy.

In the study by Magaton et al.,^(12 )^ there was elevation of the BP and a reduction in glomerular filtration rate in 3-month-old in male rats that were offspring of diabetic rats. In this study, there was no evidence of a reduction in number of nephrons, but there was early glomerular hypertrophy, which was not confirmed at our evaluation of the rats with the same age. The male offspring rats from diabetic mothers had high BP in this way, it is possible that the high pressure levels are transmitted to the glomeruli, causing hypertrophy. In the female offspring of diabetic mothers, the BP values were not as elevated and therefore, at 3 months of age, they did not alter glomerular morphology. However, it is possible that with age the changes are exacerbated and the morphologic analysis will show some modifications, such as increased glomerular area and arteriolar hypertrophy.

Analysis of the BP variations during pregnancy in both groups showed that the control rats had significantly lower values than those seen in the pups of diabetic mothers. According to Ferreira et al.,^(13 )^ pregnancy is associated with systemic and intrarenal vasodilating factors, which compensate the gestational vasoconstriction factors. Their study identified an increase in levels of iNOS (inducible nitric oxide synthase), increased expression of the angiotensin II AT2 receptor and of the relaxin receptor LGR7. In the male pups of diabetic mothers, a smaller capacity for nitric oxide synthesis was noted.^(8) ^It is also possible that in the females there might be a deficit in production of this vasodilator, resulting in higher values of BP nevertheless, other experiments are needed to confirm this hypothesis.

Over the last 20 years, there has been an increase in hypertensive disorders during gestation. This might be associated, at least in part, to the increase in obesity and the tendency to have children at a later age. On the other hand, pregnancy requires a complex adaptation of the cardiovascular system due to the decrease in vascular resistance and the increase in blood volume, besides the other preexisting alterations that can complicate these adaptations. Therefore, knowledge of the predisposition to develop arterial hypertension and of the renal dysfunction in women who are children of diabetic mothers is of great importance. Although it is not appropriate to transpose the results obtained experimentally to the human species, this study aids us understand the repercussions of diabetes on offspring during pregnancy.^(14 )^

As to the kidney function parameters, it was observed that the females, as well as the males, presented with a certain degree of renal dysfunction, which might worsen the renal damage when associated with elevated BP. Data from the literature suggest that the renal dysfunction in offspring of diabetic mothers may also be associated with abnormalities of the renin-angiotensin system,^(12,15) ^as well as alterations in the sympathetic renal activation.^(16) ^Other mechanisms may also contribute towards elevation of the BP in this experimental model.

## CONCLUSION

Females subjected to intrauterine hyperglycemia show elevated pressure levels, as well as the male offspring, but to a lesser degree than the males, in addition to a certain degree of renal dysfunction. These results, along data from the literature, reinforce the need for rigorous follow-up of hyperglycemia during gestation, seeking to prevent the alterations observed experimentally in the offspring of diabetic mothers.
