# Supplementary material for: Effect of maternal diabetes on female offspring
Source: Einstein (Sao Paulo). 2014 Oct-Dec;12(4):413–9. doi: 10.1590/S1679-45082014AO3200 (PMC4879905; doi:10.1590/S1679-45082014AO3200)
Supplement: Translation

# Translation: Efeito do diabetes materno na prole feminina

**Authors:** Juliana de Oliveira Martins, Maurício Isaac Panício, Marcos Paulo Suehiro Dantas, Guiomar Nascimento Gomes

**Affiliations:** 1 Universidade Federal de São Paulo, São Paulo, SP, Brasil.

**Keywords:** Prenhez, Desenvolvimento fetal, Diabetes mellitus experimental, Hipertensão, Ureia, Creatinina, Estreptozocina/administração & dosagem

## Abstract

**Objetivo:**

Avaliar o efeito do diabetes materno sobre a pressão arterial e a função renal da prole feminina, bem como verificar se as alterações observadas se exacerbam durante a prenhez.

**Métodos:**

O *diabetes mellitus* foi induzido em ratas com a administração de estreptozocina em dose única, uma semana antes do cruzamento. Durante a prenhez, foram feitas medidas da pressão arterial por pletismografia. No 20^o^dia da prenhez, os animais foram colocados durante 24 horas em gaiolas metabólicas para obtenção de amostras de urina. Após a retirada dos animais das gaiolas, foram obtidas amostras de sangue. Um mês após a prenhez, foram obtidas novas amostras de sangue e urina para as determinações. A função renal foi avaliada por meio de proteinúria, ureia plasmática, creatinina plasmática, carga excretada de creatinina, fluxo urinário e *clearance* de creatinina.

**Resultados:**

As fêmeas da prole de mães diabéticas apresentaram elevação da pressão arterial e redução do ritmo de filtração glomerular em relação ao grupo controle.

**Conclusão:**

A hiperglicemia durante a gestação foi capaz de causar elevação da pressão arterial e disfunção renal na prole de sexo feminino.

## INTRODUÇÃO

Modificações durante o desenvolvimento embrionário têm sido associadas ao desenvolvimento de hipertensão arterial e de alterações na função renal em modelos experimentais, em que algumas alterações, como desnutrição intrauterina, indução de *diabetes mellitus* e restrição de sono, foram impostas durante o período de prenhez.^(1 - 3)^

Em modelos experimentais que mimetizam o diabetes gestacional ou o diabetes tipo 1, verificou-se que os filhos de mães diabéticas, quando adultos, apresentam hipertensão arterial e menor taxa de filtração glomerular, bem como reduzida capacidade em excretar sódio, quando submetidos a uma sobrecarga desse íon.^(2, 4 - 6)^Além disso, o estudo do córtex renal apontou aumento da expressão de canais de sódio epiteliais (ENaC) e sódio/potássio ATPase (Na^+^/K^+^ATPase), sem redução de trocador sódio/hidrogênio (NHE3) ou outros transportadores de sódio, sugerindo que a retenção de sódio deveu-se à reabsorção aumentada em segmentos distais do néfron. Recentemente, Aceti et al.^(7)^realizaram uma revisão sistemática e metanálise de estudos relatando hipertensão arterial em crianças nascidas de mães diabéticas e confirmaram que filhos de mães diabéticas têm pressão arterial (PA) maior do que os controles.

Ainda em modelos experimentais, demonstrou-se que a exposição à hiperglicemia durante o período fetal prejudica a nefrogênese.^(4)^Estudos anteriores de nosso laboratório mostraram que filhotes machos de ratas induzidas ao *diabetes mellitus* desenvolvem hipertensão arterial precoce, sem a alteração do número de néfrons. Nesses animais, a diminuição da resposta vasodilatadora dependente de endotélio observada no leito mesentérico parece contribuir para o desenvolvimento da hipertensão arterial. A suplementação dos animais com L-arginina foi capaz de prevenir o desenvolvimento da hipertensão, confirmando a participação do sistema do óxido nítriconesse modelo experimental.^(8)^Entretanto, a maior parte desses estudos foi realizada em animais do sexo masculino.

O aumento da PA é o principal fator de risco para doenças cardíacas, acidente vascular cerebral e doenças renais. Estima-se que uma em cada seis pessoas no mundo seja hipertensa e espera-se que esse número aumente para 1,5 bilhão em 2025.^(9)^No Brasil, a prevalência da hipertensão está entre 5 e 30%, dependendo da área estudada.^(10)^Embora a prevalência da hipertensão arterial seja maior nos homens, a prevalência em mulheres não é insignificante. Acredita-se que cerca de 30% das mulheres da população mundial sejam hipertensas.^(11)^O aumento da obesidade em mulheres jovens nos países desenvolvidos e em alguns países em desenvolvimento também é um dado preocupante, uma vez que deve contribuir para o aumento da incidência de diabetes em mulheres grávidas.

## OBJETIVO

Avaliar se fêmeas provenientes da prole de mães diabéticas apresentaram alterações da função renal e hipertensão arterial, além de verificar se essas alterações se acentuam durante a prenhez.

## MÉTODOS

Machos e fêmeas Wistar, obtidos no Centro de Desenvolvimento de Modelos Experimentais para Medicina e Biologia da Universidade Federal de São Paulo (UNIFESP), foram alojados em gaiolas plásticas coletivas e mantidos com livre acesso a ração e água. Para obtenção dos filhotes de mães diabéticas, fêmeas Wistar foram induzidas ao *diabetes mellitus* com a administração de estreptozotocina em dose única (60mg/kg ip.) 1 semana antes do cruzamento. Dois dias após a administração de estreptozocina, a glicemia foi determinada com o aparelho *Advantage* II (Boehringer, Mannheim, Alemanha) do sangue da veia caudal. Consideraram-se diabéticas as ratas cujos valores de glicemia foram ≥250mg/dL. Após o nascimento dos filhotes controles e de mães diabéticas, as proles foram reduzidas a seis filhotes, que permaneceram com as mães até o desmame (aos 28 dias). Após essa etapa, os filhotes foram colocados em gaiolas coletivas. Aos 2 meses e 15 dias de idade, os animais foram treinados para entrar no cilindro de contenção para as medidas de PA pelo método indireto de pletismografia. Após a obtenção de valores estáveis de PA, as fêmeas foram colocadas para cruzar. O dia 1 de prenhez foi considerado como o dia em que a prenhez foi confirmada por meio do esfregaço vaginal. Os valores de PA também foram obtidos ao longo da prenhez.

No 20º dia da prenhez, os animais foram colocados durante 24 horas em gaiolas metabólicas (Criffa, Barcelona, Espanha), para obtenção de amostras de urina, para a determinação da excreção de creatinina e proteinúria. Após a retirada dos animais das gaiolas metabólicas, foram obtidas amostras de sangue para dosagem de creatinina e ureia. Um mês após o nascimento das proles, as ratas foram colocadas novamente em gaiola metabólicas para novas determinações da função renal e, posteriormente, sacrificadas.

Este trabalho foi realizado no período de julho de 2013 a julho de 2014, com a aprovação número 0048/12 do Comitê de Ética em Pesquisa da UNIFESP. O Termo de Consentimento Livre Esclarecido não se aplicou.

### Estudo da função renal

Para o estudo da função renal, foram avaliadas as concentrações plasmáticas de ureia e de creatinina, e a concentração urinária de creatinina. Foi calculado o *clearance* de creatinina como um índice do ritmo de filtração glomerular. As concentrações plasmáticas e urinárias de creatinina foram determinadas por método colorimétrico. Para o cálculo do *clearance* usamos a fórmula:



Onde Cl indica *clearance* de creatinina; U, concentração urinária de creatinina; P, concentração plasmática de creatinina; e V, fluxo urinário.

O método utilizado para a determinação da proteinúria foi o de precipitação com ácido sulfossalicílico.

### Estudo morfológico

Os rins de fêmeas controles e filhotes de mães diabéticas não prenhez aos 3 meses foram dissecados, limpos de tecido conectivo, pesados, cortados longitudinalmente e fixados em Bouin. Foram feitos cortes histológicos de 5μm, os quais foram corados com técnica de hematoxilina e eosina e Verhoeff. As imagens foram adquiridas em microscópio Nikon^®^conectado a um microcomputador. A análise das imagens foram feitas em simples cego.

Em cada lâmina corada pela hematoxilina e eosina foram obtidas 20 imagens (campos) sequenciais, iniciando a partir do hilo e percorrendo o córtex renal, sendo analisados a área glomerular e o número de glomérulos por campo.

A fim de avaliar a espessura arteriolar, em cada lâmina corada por Verhoeff, foram analisadas 12 arteríolas, das quais se obtiveram 4 medidas de espessura por arteríola.

### Análise estatística

Para a análise estatística, empregaram-se os testes *t* não pareado e de Análise de Variância *one-way* ( *one-way* ANOVA), seguidos pelo teste Bonferroni. Foi definido como estatisticamente significante p≤0,05. Os resultados foram apresentados como média±erro padrão.

## RESULTADOS

A tabela 1 apresenta os valores médios da pressão arterial sistólica medidos ao longo do período de prenhez nos grupos controle e de filhotes de mães diabéticas, como também a média da PA anterior a esse período ( Figura 1 ).

**Tabela 1:** Valores médios de pressão arterial sistólica (em mmHg) medidas ao longo do período de experimentação nos grupos controle e de filhotes de mães diabéticas

Dia de prenhez	Controle	Filhotes de mães diabéticas
Antes da prenhez	119,98±1,07 (44/11)	127,39±1,09 (55/13)
Dia 1	125,97±2,40 (33/11)	130,85±1,98 (38/10)
Dia 2	123,92±1,65 (24/7)	131,90±2,51 (40/10)
Dia 3	122,00±4,08 (7/2)	128,25±5,12 (20/5)
Dia 4	131,63±3,62 (8/2)	124,90±1,33 (8/2)
Dia 5	126,50±1,28 (26/8)	134,68±1,47 (20/5)
Dia 6	123,44±2,38 (27/8)	131,97±2,00 (39/11)
Dia 7	119,17±2,06 (30/10)	134,42±1,86 (39/10)
Dia 8	116,75±1,45 (32/10)	132,96±2,00 (30/10)
Dia 9	118,72±1,93 (25/7)	127,67±2,28 (26/7)
Dia 10	115,00±3,03 (12/4)	133,67±2,35 (21/6)
Dia 11	118,93±3,58 (14/4)	130,84±3,63 (15/4)
Dia 12	117,00±1,99 (27/8)	125,70±2,18 (31/8)
Dia 13	119,00±2,27 (22/7)	122,26±2,21 (37/10)
Dia 14	115,76±1,64 (29/8)	132,06±1,79 (35/10)
Dia 15	117,21±2,66 (34/10)	128,52±1,70 (40/11)
Dia 16	113,84±2,80 (19/6)	133,52±1,41 (27/10)
Dia 17	102,67±4,33 (3/1)	130,17±2,00 (14/7)
Dia 18	115,18±3,18 (11/3)	120,13±4,19 (12/4)
Dia 19	110,93±2,10 (28/8)	118,88±3,68 (16/7)
Dia 20	109,84±2,06 (25/7)	127,39±6,67 (12/6)
Média geral	118,26	128,91

Valores apresentados como média±erro padrão (número de medidas/número de animais).

**Figura 1:** Pressão arterial sistólica (em mmHg) ao longo do período de prenhez em grupos controle e de filhotes de mães diabéticas. Valor de p≤0,05 *versus* C

Os parâmetros de função renal ao 20º dia de prenhez mostraram que o grupo de filhotes de mães diabéticas apresentou redução do *clearance* de creatinina e aumento da concentração plasmática de ureia, contudo não houve redução significante da carga excretada de creatinina ( Figura 2 e Tabela 2 ).

**Figura 2:** Função renal de ratas controles e filhotes de mães diabéticas ao 20 o dia de prenhez. (A) Carga excretada de creatinina; (B) *clearance* de creatinina; (C) concentração de ureia no plasma. *Valor de p≤0,05 *versus* C

**Tabela 2:** Parâmetros de função renal no 20 o dia da prenhez

	Proteinúria (mg/24h)	Ureia plasmática (mg/dL)	Creatinina plasmática (mg/mL)	Carga excretada de creatinina (mg/24h)	*Clearance* de creatinina (mL/min)	Fluxo urinário (mL/24h)
Controles	2,17±0,38 (11)	37,09±3,78 (13)	1,20±0,25 (10)	8,96±1,05 (15)	0,86±0,19 (10)	11,70±0,87 (15)
Filhotes de mães diabéticas	1,95±0,37 (11)	47,26±1,9* (14)	1,02±0,10 (9)	5,93±0,87 (10)	0,37±0,07* (8)	11,90±0,62 (15)

*Valor de p≤0,05 *versus* controle. Valores apresentados como média ± erro padrão (n).

A reavaliação da função renal após um mês também demonstrou redução do *clearance* de creatinina em fêmeas filhotes de mães diabéticas ( Figura 3 e Tabela 3 ).

**Figura 3:** Função renal de ratas controles e filhotes de mães diabéticas 1 mês após período de prenhez. (A) Carga excretada de creatinina; (B) *clearance* de creatinina. *Valor de p≤0,05 *versus* controle

**Tabela 3:** Parâmetros de função renal 1 mês após a prenhez

	Proteinúria (mg/24h)	Ureia plasmática (mg/dL)	Creatinina plasmática (mg/dL)	Carga excretada de creatinina (mg/24h)	*Clearance* de cretinina (mL/min)	Fluxo urinário (mL/24h)
Controles	0,93±0,28 (9)	53,85±5,84 (12)	0,74±0,13 (12)	9,12±1,02 (12)	1,09±0,19 (12)	11,99±0,99 (12)
Filhotes de mães diabéticas	0,96±0,27 (11)	41,33±1,95 (12)	0,86±0,07 (12)	6,98±1,02 (12)	0,63±0,11* (12)	11,50±0,84 (12)

*Valor de p≤0,05 *versus* controle. Valores apresentados como média ± erro padrão (n).

Comparando ratas controles e filhotes de mães diabéticas não prenhez aos 3 meses, foi observado um comprometimento da função renal evidenciado pelo aumento da concentração plasmática de ureia, o qual, como vimos anteriormente, exacerba-se com o estado de prenhez (Figuras 4A, 4B, 4C e Tabela 4 ).

**Tabela 4:** Parâmetros de função renal de ratas não prenhez aos 3 meses

	Proteinúria (mg/24h)	Ureia plasmática (mg/dL)	Creatinina plasmática (mg/dL)	Carga excretada de creatinina (mg/24h)	*Clearance* de cretinina (mL/min)	Fluxo urinário (mL/24h)
Controles	1,17±0,28 (4)	31,45±2,10 (8)	0,80±0,15 (5)	8,61±1,24 (8)	0,94±0,13 (5)	7,96±1,09 (9)
Filhotes de mães diabéticas	0,68±0,11 (2)	46,98±4,7* (6)	0,67±0,08 (6)	7,76±0,48 (7)	0,86±0,09 (6)	9,50±0,51 (7)

*Valor de p≤0,05 *versus* controle. Valores apresentados como média±erro padrão (n).

A análise da morfometria renal em hematoxilina e eosina não mostrou diferença estatística em área glomerular e nos números de glomérulos nessas ratas aos 3 meses ( Figuras 4D, 4E e 4F ). A avaliação da espessura arteriolar em Verhoff também não revelou diferença entre os grupos ( Figura 4G e 4H ).

**Figura 4:** Ratas controles e filhotes de mães diabéticas não prenhez aos 3 meses. Função renal: (A) carga excretada de creatinina; (B) *clearance* de creatinina; (C) ureia plasmática. Morfologia renal: (D) área glomerular; (E) número de glomérulos por campo adquirido; (F) fotomicrografias de cortes histológicos em hematoxilina e eosina; (G) espessura arteriolar; (H) fotomicrografias de cortes histológicos em Verhoeff. *p≤0,05 *versus* controle

## DISCUSSÃO

Observou-se, no presente trabalho, que a prole feminina desenvolveu pré-hipertensão e, mesmo não apresentando elevados níveis pressóricos como a prole do sexo masculino, as ratas expostas à hiperglicemia intraútero já apresentaram alterações precoces da função renal, as quais se tornam mais evidentes com o estado de prenhez.

No estudo de Magaton et al.,^(12)^houve elevação da PA e redução do ritmo de filtração glomerular em ratos machos de 3 meses da prole de ratas diabéticas. Neste trabalho, não se evidenciou redução do número de néfrons, mas houve hipertrofia glomerular precoce, a qual não se confirmou em nossa avaliação nas ratas de mesma idade. Os ratos machos filhotes de mães diabéticas apresentaram PA elevada; desse modo, é possível que os elevados níveis pressóricos sejam transmitidos aos glomérulos, causando hipertrofia. Nas fêmeas filhotes de mães diabéticas, os valores de PA não foram tão elevados e, por isso, aos 3 meses de idade, não alteraram a morfologia glomerular. Entretanto, é possível que, com o envelhecimento, as alterações sejam exacerbadas e a análise morfológica demonstre modificações, como o aumento da área glomerular e hipertrofia arteriolar.

A análise das variações de PA durante a prenhez nos dois grupos experimentais mostrou que as ratas controles apresentam valores significativamente menores que os observados no grupo de filhotes de mães diabéticas. Segundo Ferreira et al.,^(13)^a gestação está associada com fatores vasodilatadores sistêmicos e intrarrenais, que compensam os fatores gestacionais vasoconstritores. Em seu estudo, foram identificados aumento dos níveis de iNOS ( *inducible nitric oxide synthase* ), aumento da expressão do receptor AT2 de angiotensina II e do receptor LGR7 de relaxina. Nos filhotes machos de mães diabéticas, observou-se menor capacidade de síntese de óxido nítrico.^(8)^É possível que também nas fêmeas haja um défice na produção desse vasodilatador, resultando em valores mais elevados de PA; entretanto, outros experimentos são necessários para confirmar essa hipótese.

Nos últimos 20 anos, houve um aumento na incidência de desordens hipertensivas durante a gestação. Isso pode ser, pelo menos em parte, associado ao aumento da obesidade e pela tendência de ter filhos mais tardiamente. Por outro lado, a gravidez exige uma adaptação complexa do sistema cardiovascular frente à diminuição da resistência vascular e ao aumento do volume sanguíneo, além de outras alterações preexistentes, que podem complicar essas adaptações. Assim, o conhecimento da predisposição para desenvolver hipertensão arterial e da disfunção renal em mulheres filhas de mães diabéticas é de grande importância. Embora não seja adequado transpor os resultados obtidos experimentalmente para a espécie humana, este estudo nos auxilia no conhecimento da repercussão do diabetes na prole durante a prenhez.^(14)^

Quanto aos parâmetros de função renal, observou-se que as fêmeas, assim como os machos, apresentam certo grau de disfunção renal que, associado à elevação da PA, pode vir a agravar o dano renal. Dados da literatura sugerem que a disfunção renal em filhotes de mães diabéticas também pode estar associada a anormalidades do sistema renina-angiotensina,^(12, 15)^bem como a alterações na ativação simpática renal.^(16)^Outros mecanismos também podem contribuir para a elevação da PA neste modelo experimental.

## CONCLUSÃO

Fêmeas submetidas à hiperglicemia intraútero apresentaram elevação dos níveis pressóricos, assim como a prole masculina, todavia, em menor grau que os machos, além de certo grau de disfunção renal. Estes resultados, em conjunto com os dados da literatura, reforçam a necessidade de um acompanhamento rigoroso da hiperglicemia durante a gestação, visando prevenir as alterações observadas experimentalmente na prole de mães diabéticas.
